# Clinical incidence and relevance of incomplete endothelialization in atrial fibrillation patients with Left Atrial Appendage Closure

**DOI:** 10.1186/s12872-024-04113-5

**Published:** 2024-08-23

**Authors:** Jini Zhu, Yanpeng Wang, Meifang Li, Dong Huang, Shuai Li, Jingbo Li

**Affiliations:** 1 Department of Cardiology, Shanghai Sixth People’s Hospital, Shanghai Jiao Tong University School of Medicine, Shanghai, China; 2 Department of Emergency, Shanghai Sixth People’s Hospital, Shanghai Jiao Tong University School of Medicine, Shanghai, China

**Keywords:** Atrial fibrillation, Left atrial appendage closure, Incomplete device endothelialization, Oral anticoagulants

## Abstract

**Background:**

The objective of this study is to investigate the incidence, potential risk factors, and clinical outcomes of incomplete device endothelialization (IDE) in atrial fibrillation (AF) patients undergoing Watchman left atrial appendage closure (LAAC).

**Methods:**

In this study, 68 AF patients who underwent successful implantation of the Watchman device without peri-device leak (PDL) during follow-up were included. The endothelialization status was assessed using Transesophageal echocardiography (TEE) and LAA computed tomography angiography (CTA) at 6 weeks and 6 months post-implantation. Adverse cerebro-cardiac events were documented at one-year follow-up. Baseline characteristics, including age, device sizes, and clinical indicators, were analyzed as potential predictors for IDE.

**Results:**

IDE was observed in 70.6% and 67.6% of patients at 6 weeks and 6 months after implantation, respectively. Higher levels of high-density lipoprotein cholesterol (HDL-C) [odds ratio (OR): 15.109, 95% confidence interval (CI): 1.637-139.478, *p* = 0.017 and OR: 11.015, 95% CI: 1.365–88.896, *p* = 0.024] and lower aspartate aminotransferase (AST) (OR 0.924, 95% CI: 0.865–0.986, *p* = 0.017 and OR: 0.930, 95% CI: 0.874–0.990, *p* = 0.023) at baseline were found to be significantly associated with IDE at 6 weeks and 6 months, respectively, although no significant difference in adverse cerebro-cardiac events was noted between incomplete and complete DE groups during 1-year follow-up

**Conclusions:**

IDE is found to be a prevalent occurrence in humans following LAAC. Elevated HDL-C and reduced AST levels are shown to be linked to an increased risk of IDE after LAAC

## Introduction

Percutaneous left atrial appendage closure (LAAC) has emerged as effective alternative to long-term oral anticoagulants (OAC) for stroke prevention, especially in patients with nonvalvular atrial fibrillation (NVAF) [1]. Following percutaneous LAAC, the implanted device is susceptible to thrombus formation on the exposed fabric and metal surfaces that come into contact with blood on the atrial side [2, 3]. Consequently, an antithrombotic regimen is applied to prevent device-related thrombosis (DRT) until the device’s surface achieves complete endothelialization. However, current anticoagulation and antiplatelet strategies post LAAC are largely empirical, relying on findings from animal studies [4]. According to these studies, complete device endothelialization (CDE) typically occurs within 45–90 days [4, 5]. As a result, human antithrombotic therapy is recommended for a similar duration [6].

In practice, incomplete device endothelialization (IDE) has been frequently reported in humans at 6 months or later after implantation [7–10]. This necessitates investigation into the risk factors and early detection methods for adjusting antithrombotic strategies. Additionally, there is a paucity of studies examining the impact of IDE on adverse outcomes in Chinese patients. Therefore, this study was conducted to assess the prevalence of IDE at 6 weeks and 6 months post Watchman LAAC using both Transesophageal echocardiography (TEE) and LAA computed tomography angiography (CTA) at follow-up. The study also aimed to investigate potential predictors of IDE and evaluate its effects on adverse cerebro-cardiac events in Chinese AF patients undergoing LAAC.

## Methods

### Study design

A total of 98 Chinese patients with AF, who underwent successful LAAC using the Watchman device (Boston Scientific Corporation; Natick, MA, USA) at our institution between April 2020 and April 2022, were consecutively enrolled. Inclusion criteria comprised age > 18 years, symptomatic NVAF refractory to antiarrhythmic drugs, and a CHA2DS2-VASc Score ≥ 2, along with one of the following situations: (1) high bleeding risk (HAS-BLED Score ≥ 3); (2) history of stroke or systemic embolic event despite OAC treatment; (3) intolerance to chronic OAC due to minor bleeding from anticoagulation therapy; and (4) preference for LAAC device implantation as an alternative to long-term OAC despite adequate information. Exclusion criteria included mechanical heart valve, left ventricular ejection fraction (LVEF) < 30%, intracardiac thrombus, and end-stage renal disease [estimated glomerular filtration rate (eGFR) < 15 mL/min/1.73 m²]. All Watchman device implantations were performed by the certified physician (Jing-Bo Li) who had undergone rigorous training to ensure expertise. Thirty participants were excluded due to peri-device leakage (PDL, *n* = 16) during follow-up, inability to perform TEE or LAA CTA (*n* = 4), loss of follow-up (*n* = 6), and incomplete the planned medical therapies (*n* = 2), lack of data (*n* = 2). Finally, the remaining 68 participants were entered into our analysis, who were identified as non-PDL after performing both TEE and LAA CTA at follow-up.

The study received approval from the ethics committee of Shanghai Sixth People’s Hospital affiliated with Shanghai Jiao Tong University School of Medicine, and written informed consents were obtained from all participants.

### Physical examinations and laboratory measurements

Physical and laboratory examinations were conducted by trained physicians. During a standard interview upon admission to the Cardiology Department, information on height, weight, and medical history, including diabetes and hypertension, was collected. Body mass index (BMI) was calculated as weight divided by the square of height. Blood indicators such as white blood cell (WBC) count, hemoglobin, alanine aminotransferase (ALT), aspartate transaminase (AST), serum creatinine (SCr), estimated glomerular filtration rate (eGFR), serum uric acid (SUA), total cholesterol (TC), total triglyceride (TTG), high-density lipoprotein cholesterol (HDL-C), low-density lipoprotein cholesterol (LDL-C), fasting blood glucose (FPG), and N-terminal pro-B-type natriuretic peptide (NT-proBNP) were obtained from blood samples after an overnight fast within 24 h after admission and before LAAC.

### LAAC Procedure and patient follow-up

**Fig. 1:**
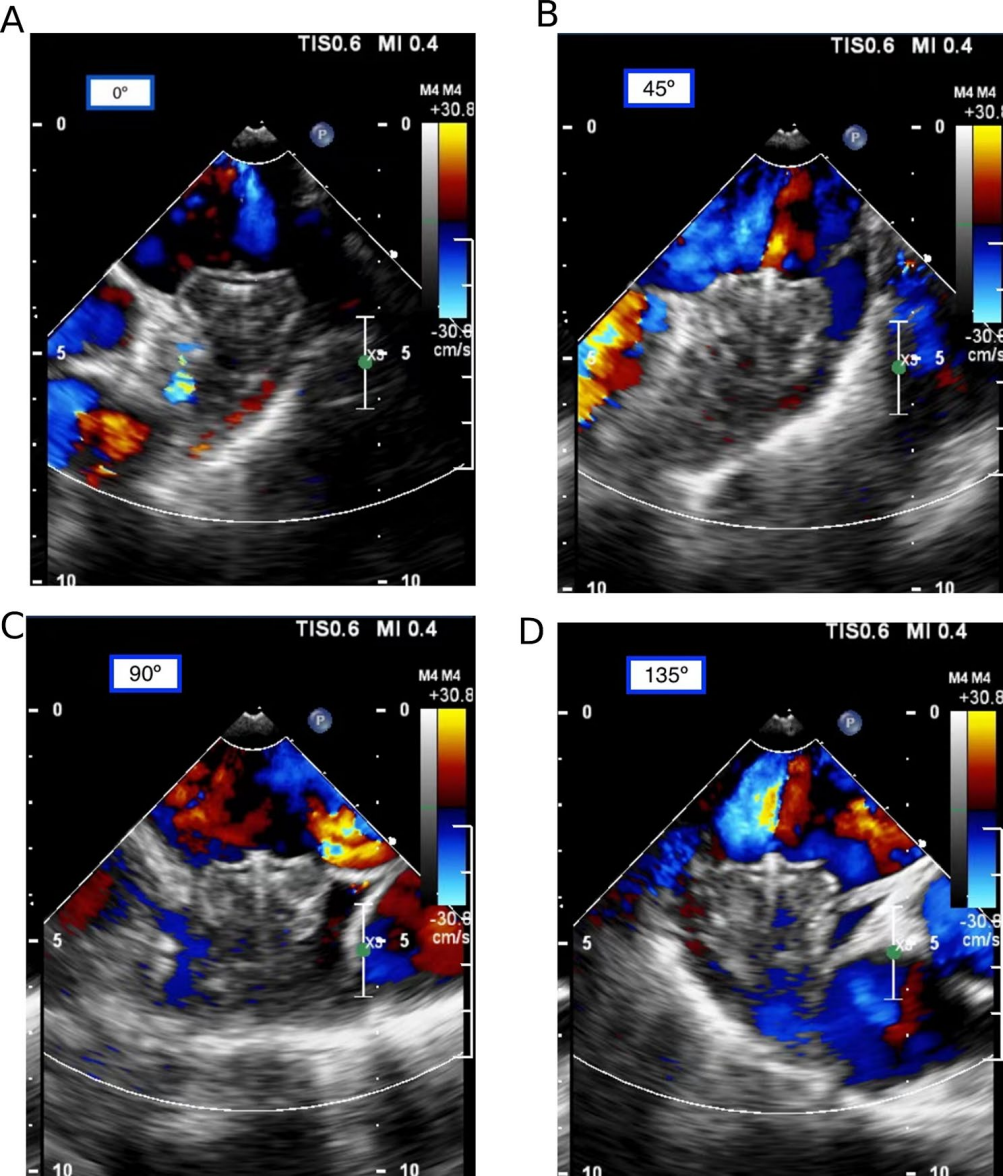
**A-D** One case (a 60-year-old woman with persistent atrial fibrillation) without PDL after LAAC. After Watchman device was implanted, TEE showed that no peri-device flow at 0, 45, 90, and 135 °C of LAA section, indicating complete seals without PDL.

All patients underwent Watchman LAAC device implantation following a standardized protocol previously described [11, 12]. The device was implanted using TEE, LAA angiography, and fluoroscopic guidance through the right femoral vein and transseptal access. TEE and LAA angiography were employed to rule out LAA thrombosis, and the type and size of LAAC devices were chosen based on LAA orifice, diameter, depth, and morphology [13]. Following the procedure, all implants met the PASS criteria for complete seals [2]. Subsequently, all patients received 45 days anticoagulation, followed by dual antiplatelet therapy (DAPT) for 6 months, and then lifelong aspirin, as per the PROTECT-AF trial [2]. TEE and LAA CTA follow-ups were scheduled at 6 weeks and 6 months post-implantation to identify DRT or PDL. PDL, due to allowing residual flow in the LAA regardless of neoendothelialization, was excluded from this study (Fig. 1). Data regarding NYHA class, medications at discharge, and adverse clinical events were obtained from discharge summaries. After hospital discharge, patients were followed up concerning anticoagulant therapies and adverse clinical events for 12 months.

### Diagnostic criteria and outcomes

**Fig. 2:**
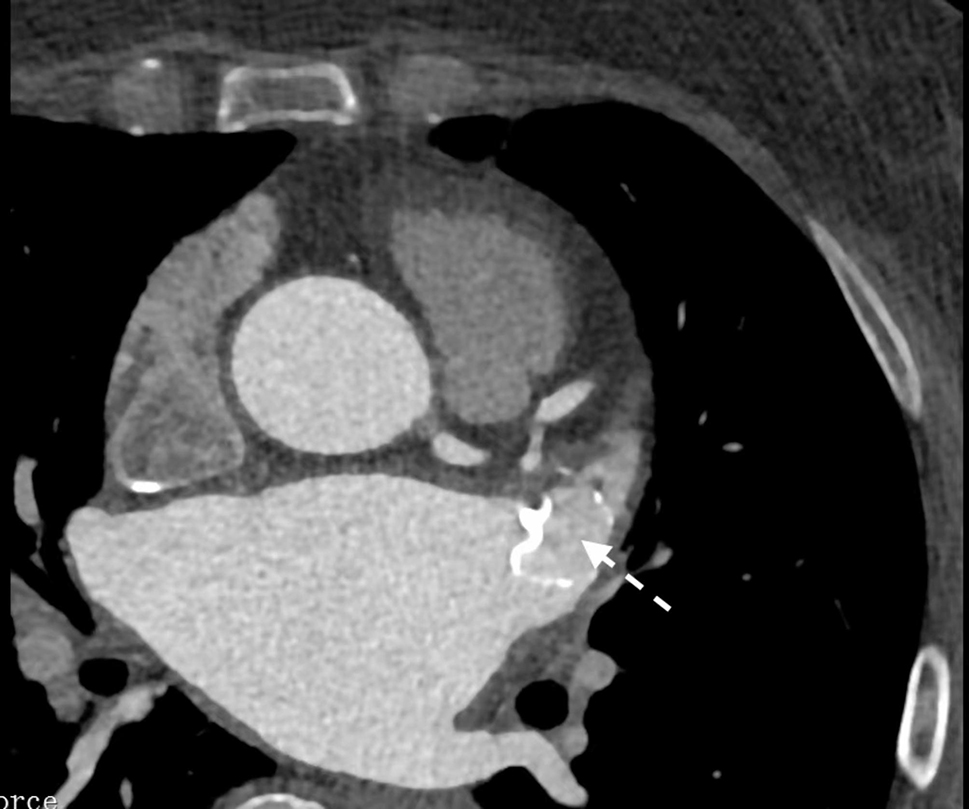
One case (a 60-year-old woman with persistent atrial fibrillation) for IDE at 6 weeks follow-up after LAAC. LAA CTA showed residual contrast agent flow inside the LAA (as shown by arrow)

**Fig. 3:**
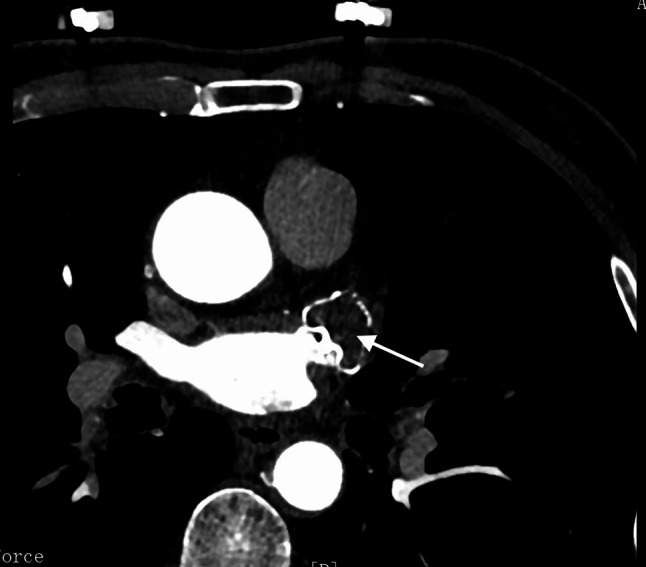
One case (a 72-year-old man with persistent atrial fibrillation) for CDE at 6 weeks follow-up after LAAC. LAA CTA showed no residual contrast agent flow inside the LAA (as shown by arrow)

CHA2DS2-VASc and HAS-BLED scores were computed using published literature [14, 15]. LAAC was defined as the satisfactory positioning of the device at the ostium, covering all trabeculated portions of the LAA, with peri-device flow < 5 mm [16]. IDE was defined as residual contrast agent flow inside the LAA without PDL at 6 weeks or 6 months follow-up (Fig. 2) [17]. The absence of both residual flow and PDL was defined as CDE at 6 weeks or 6 months follow-up (Fig. 3) [17]. DRT was defined as the detection of a thrombus adherent to the luminal (left atrial) side of the device by senior ultrasound physicians through TEE scan [18, 19]. Relevant adverse cerebro-cardiac events were assessed at the 1-year follow-up post-LAAC, including all-cause death, acute ischemic stroke (AIS), transient ischemic attack (TIA), peripheral vascular embolism, heart failure (HF) hospitalization, and major bleeding (Bleeding Academy Research Consortium bleeding score > 3 points) [20].

### Statistical analyses

Data were subjected to analysis using SPSS 19.0 software. The sample size was calculated at least 97 cases using PASS software based on the study of Simard et al. [21]. Initially, the Shapiro-Wilk test was employed to assess the normality of continuous variables. Normally distributed variables were presented as mean ± standard deviation and compared using an independent t-test, while non-normally distributed variables were described as median with interquartile range (IQR) and compared using the Mann-Whitney U test. Subsequently, categorical variables were expressed as absolute numbers (percentages) and compared using the χ^2^ test. Univariable, age- and sex-adjusted, and multivariable logistic regression analyses were conducted to identify predictors of IDE. Variables with *p* < 0.05 in univariate analysis were included in the multivariate model through forward stepwise regression. A significance level of *p* < 0.05 was considered statistically significant.

## Results

### Baseline characteristics of studied subjects

Among the 68 participants analyzed, the mean age at the time of consent was 71 ± 9 years (range 53–89), with 52.9% being male. The stroke risk scores for CHADS2 and CHA2DS2-VASc were 2.3 ± 1.3 and 4.3 ± 1.5, respectively, while the HAS-BLED score was 2.0 ± 0.8. Remarkably, at 6 weeks and 6 months after LAAC, 70.6% and 67.6% of patients were found to have IDE, respectively.

Table 1 displays the baseline demographic and clinical characteristics between IDE and CDE at 6 weeks and 6 months post-LAAC, respectively. Regardless of 6 weeks or 6 months post-LAAC, patients with IDE exhibited higher levels of HDL-C compared to those with CDE (1.23 ± 0.31 vs. 1.03 ± 0.35 mmol/L for 6 weeks and 1.24 ± 0.31 vs. 1.05 ± 0.33 mmol/L for 6 months; both *p* < 0.05). However, no significant differences were observed in terms of age, gender, BMI, history of disease risk factors, AF pattern, and other laboratory indicators between the two groups.

**Table 1:** Clinical characteristics of the subjects

Variables	Total population (*n* = 68)	6 weeks post-LAAC				6 months post-LAAC					
		CDE (*n* = 20)	IDE (*n* = 48)	*p* value		CDE (*n* = 22)	IDE (*n* = 46)			*p*’ value	
Age (years)	71 ± 9	70 ± 9	71 ± 9	0.842		71 ± 9	71 ± 9			0.877	
Men (n, %)	36(52.9%)	12(60%)	24(50%)	0.452		13(59.1%)	23(50%)			0.482	
BMI (kg/m^2^)	25.47 ± 3.77	25.6 ± 3.78	25.43 ± 3.82	0.886		25.41 ± 3.65	25.49 ± 3.87			0.943	
CHADS_2_ score	2.3 ± 1.3	2.4 ± 1.3	2.3 ± 1.4	0.720		2.3 ± 1.3	2.3 ± 1.4			0.969	
CHA_2_DS_2_-VASc score	4.3 ± 1.5	4.1 ± 1.5	4.4 ± 1.6	0.447		4.1 ± 1.4	4.4 ± 1.6			0.396	
HAS-BLED score	2.0 ± 0.8	2.0 ± 0.6	2.0 ± 0.9	0.852		2.0 ± 0.7	2.0 ± 0.9			0.913	
AF pattern (n, %)				0.820						0.472	
Paroxysmal	27(39.7%)	9(45%)	18(37.5%)			11(50)	16(34.8)				
Persistent	38(55.9%)	10(50%)	28(58.3%)			10(45.5)	28(60.9)				
Long-standing persistent	3(4.4%)	1(5%)	2(4.2%)			1(4.5)	2(4.3)				
Previous history											
CHF (n, %)	4(5.9%)	0(0)	4(8.3%)	0.183		0(0)	4(8.7%)			0.154	
CAD (n, %)	14(20.6%)	3(15%)	11(22.9%)	0.462		3(13.6%)	11(23.9%)			0.327	
Hypertension (n, %)	56(82.4%)	17(85%)	39(81.3%)	0.712		19(86.4%)	37(80.4%)			0.549	
Diabetes (n, %)	18(26.5%)	4(20%)	14(29.2%)	0.435		4(18.2%)	14(30.4%)			0.284	
History of TIA/Stroke (n, %)	19(279%)	8(40%)	11(22.9%)	0.153		8(36.4%)	11(23.9%)			0.284	
Vascular disease (n, %)	50(73.5%)	13(68.4%)	37(77.1%)	0.463		13(68.4%)	37(77.1%)			0.463	
Hyperlipemia (n, %)	17(25%)	5(25%)	12(25%)	1.000		6(27.3%)	11(23.9%)			0.765	
Cardiac pacemaker implantation (n, %)	4(5.9)	2(10%)	2(4.2%)	0.352		2(9.1%)		2(4.3%)		0.437	
Laboratory tests at admission											
WBC (×10^9^/L)	7.11 ± 2.10	6.77 ± 1.75	7.26 ± 2.24	0.383		6.83 ± 1.73		7.26 ± 2.27		0.442	
Hemoglobin (g/L)	137 ± 19	141 ± 17	135 ± 19	0.170		141 ± 16			134 ± 19	0.148	
Platelet (×109/L)	198 ± 47	207 ± 54	194 ± 43	0.283		212 ± 54			191 ± 42	0.115	
TC (mmol/L)	4.04 ± 1.09	4.11 ± 1.33	4.01 ± 1.00	0.758		4.11 ± 1.26			4.00 ± 1.02	0.713	
TTG (mmol/L)	1.43 ± 0.66	1.62 ± 0.67	1.36 ± 0.65	0.169		1.60 ± 0.67			1.35 ± 0.64	0.156	
HDL-C (mmol/L)	1.18 ± 0.33	1.03 ± 0.35	1.23 ± 0.31	0.023		1.05 ± 0.33			1.24 ± 0.31	0.031	
LDL-C (mmol/L)	2.20 ± 0.80	2.36 ± 0.99	2.14 ± 0.72	0.326		2.33 ± 0.94			2.14 ± 0.73	0.387	
*HbA1c (%)	6.1(5.7–6.6)	5.8(5.7–6.4)	6.2(5.5–6.8)	0.552		5.9(5.7–6.4)			6.1(5.5-7.0)	0.544	
*FBG (mmol/L)	5.46(4.69–6.17)	5.48(4.80–6.23)	5.30(4.69–6.30)	0.754		5.46(4.64–5.68)			5.41(4.69–6.30)	0.909	
*ALT (U/l)	28(22–39)	25(17–49)	29(24–44)	0.099		26(18–45)			29(24–44)	0.131	
*AST (U/l)	31(26–37)	34(30–40)	31(27–55)	0.057		34(31–43)			30(27–35)	0.054	
*SCr (µmol/l)	81(64–100)	78(68–99)	82(63–103)	0.893		75(63–100)			89(72–118)	1.000	
SUA (µmol/l)	409 ± 98	392 ± 100	416 ± 98	0.367		392 ± 98			417 ± 98	0.328	
eGFR (ml/min/1.73m^2^)	71.15 ± 26.61	75.56 ± 26.94	69.32 ± 26.54	0.382		74.21 ± 27.29			69.69 ± 26.46	0.517	
*NT-proBNP (ng/L)	704(306–1126)	720(276–1137)	648(306–1074)	0.891		654(249–1129)			719(306–1079)	0.728	

Values are expressed as the mean ± standard deviation, median with interquartile range, or percentages

*Non-normal distribution of continuous variables

Abbreviations: CDE, complete device endothelialization; IDE, incomplete device endothelialization; BMI, Body mass index; CHADS2 = congestive heart failure, hypertension, age ≥ 75 years, diabetes mellitus, prior stroke or transient ischemic attack; CHA2DS2-VASc = congestive heart failure, hypertension, age ≥ 75 years, diabetes mellitus, prior stroke, transient ischemic attack, or thromboembolism, vascular disease, age 65-74 years, sex category (female); HAS-BLED = hypertension, abnormal renal or liver function, stroke, bleeding, labile international normalized ratio, elderly, drugs or alcohol; AF, Atrial fibrillation; CHF, congestive heart failure; CAD, coronary artery disease; TIA, transient ischemic attack; CKD, chronic kidney disease; WBC, white blood cell; TC, Total cholesterol; TTG, Total Triglyceride; HDL-C, High-density lipoprotein cholesterol; LDL-C, Low-density lipoprotein cholesterol; HbA1c, glycosylated haemoglobin A1c; FBG, Fasting blood glucose; ALT, alanine aminotransferase; AST, aspartate transaminase; SCr, serum creatinine; SUA, serum uric acid; eGFR, estimated glomerular filtration rate; NT-proBNP, N-terminal pro-B-type natriuretic peptide

### Procedural characteristics and device outcomes for LAAC

During the procedure, the mean maximum diameter of the LAA orifice, as measured by TEE, was 22.5 ± 4.2 mm (range 16–31), resulting in a final median device size of 27 mm. The TEE measurements and the five Watchman sizes used were compared between the IDE and CDE groups, as presented in Table 2. LVEF, LA size, mitral regurgitation, maximum diameter of LAA orifice, LAA morphologies, and device size did not exhibit any significant differences between the two cohorts. Notably, there were no procedural complications or bleeding events during hospitalization, and all patients were transitioned to OAC therapy before discharge. The medical regimens at discharge, including anticoagulant and lipid-lowering medications, were similar between the two groups (Table 2).

**Table 2:** Comparison of the characteristics of LAA and LAAC procedure

Variables	6 weeks post-LAAC				6 months post-LAAC		
	CDE	IDE	*p* value		CDE	IDE	*p*’ value
TTE measurement							
LVEF (%)	60(57.5–63)	61(60–64)	0.233		60(59–64)	61(60–64)	0.233
LA size (mm)	46.5 ± 4.9	42.7 ± 6.7	0.447		46.5 ± 4.9	42.7 ± 6.7	0.447
Mitral regurgitation (n, %)			0.343				0.277
mild	11(55)	30(62.5)			12(54.5)	29(63)	
moderate	0(0)	3(6.3)			0(0)	2(6.5)	
LAA morphology (n, %)			0.085				0.204
chicken-wing	9(45)	10(20.8)			9(45)	10(20.8)	
cauliflower	9(45)	35(72.9)			11(50)	33(71.7)	
others	2(10)	3(6.3)			2(9.1)	3(6.5)	
Maximum diameter of LAA orifice (mm)	22.4 ± 3.1	22.8 ± 3.8	0.700		22.4 ± 3.0	22.8 ± 4.0	0.610
Maximum depth of LAA orifice (mm)	25.5 ± 2.6	23.5 ± 4.8	0.443		25.5 ± 2.6	23.5 ± 4.8	0.443
Maximum emptying speed of LAA (cm/s)	30.1 ± 20.8	34.1 ± 13.8	0.484		30.1 ± 20.2	34.4 ± 13.9	0.393
**Procedural characteristics of LAAC**							
Size of the Watchman™ device (n, %)			0.767				0.647
21 mm	1(5)	8(16.7)			1(4.5)	8(17.4)	
24 mm	5(25)	12(25)			6(27.3)	11(21.9)	
27 mm	7(35)	13(27.1)			8(36.4)	12(26.1)	
30 mm	4(20)	8(16.7)			4(18.2)	8(17.4)	
33 mm	3(15)	7(14.6)			3(13.6)	7(15.2)	
Watchman device size (mm)	27(24–30)	27(24–30)	0.399		27(24–30)	27(24–30)	0.531
Device compression ratio (%)	18.8(16.5–24.3)	16.7(14.9–19.8)	0.140		18.8(16.5–24)	16.7(15–20)	0.232
**Medications at discharge**							
Anticoagulation (n, %)			0.724				0.842
warfarin	1(5)	3(6.3)			1(5)	3(6.3)	
Dabigatran	5(25)	8(16.7)			5(25)	8(16.7)	
Rivaroxaban	14(70)	37(77.1)			14(70)	37(77.1)	
lipid-lowering drug (n, %)	12(60)	24(50)	0.452		13(59.1)	23(50)	0.482

Abbreviation: CDE, complete device endothelialization; IDE, incomplete device endothelialization; LAAC, left atrial appendage closure; TTE, transesophageal echocardiography; LVEF, left ventricular ejection fraction; LA, left atrium; LAA, left atrial appendage

### Clinical outcomes of 1-year follow-up

All patients successfully completed the planned 45-day regimen of anticoagulation, and no instances of DRT were observed at both the 6-week and 6-month follow-ups. Throughout the 1-year follow-up period, no deaths occurred. One patient (1.5%) experienced AIS, four patients (5.9%) suffered TIA, and two patients (2.9%) were hospitalized due to HF. No significant difference of the rate of adverse cerebro-cardiac events was observed between the IDE and CDE groups at 1-year follow-up (Table 3).

**Table 3:** Comparison of MACCE at 1-year clinical follow-up after LAAC

Variables	6 weeks post-LAAC				6 months post-LAAC		
	CDE	IDE	*p* value		CDE	IDE	*p*’ value
All-cause death, n (%)	0	0	NA		0	0	NA
acute ischemic stroke, n (%)	0(0)	1(2.1)	0.516		0	1(2.2)	0.486
transient ischemic attack, n (%)	1(5.0)	3(6.3)	0.842		2(9.1)	2(4.3)	0.437
Peripheral vascular embolism, n (%)	0(0)	0	NA		0	0	NA
HF hospitalization, n (%)	0	2(4.2)	0.354		0	2(4.3)	0.321
Major bleeding, n (%)	0	0	NA		0	0	NA
Total MACCE, n (%)	1(5)	6(12.5)	0.570		2(9.1)	5(10.9)	0.711

Abbreviation: CDE, complete device endothelialization; IDE, incomplete device endothelialization; HF, heart failure; MACCE, major adverse cardio-cerebral events

### Association of variables with IDE at follow-up

Factors associated with IDE at follow-up in three statistical models are presented in Table 4. A significant association was observed between IDE at 6-weeks follow-up and LAA morphologies (cauliflower, chicken-wing), HDL-C, ALT, and AST in the non-adjusted model (all *p* < 0.05). After adjusting for age and sex, this significant association existed in cauliflower [odds ratio (OR) 3.249, 95% confidence interval (CI) 1.056–9.990], HDL-C (OR 8.394, 95%CI 1.037–67.966), ALT (OR 0.967, 95%CI 0.936-1.000), and AST (OR 0.932, 95%CI 0.880–0.987) (all *p* < 0.05). After further controlling for other risk factors, only HDL-C (OR 15.109, 95%CI 1.637-139.478; *p* = 0.017) and AST (OR 0.924, 95%CI 0.865–0.986; *p* = 0.017) remained significant predictors of IDE at 6-weeks follow-up. Likewise, multivariate logistic regression analysis also demonstrated that HDL-C (OR 11.015, 95%CI 1.365–88.896; *p* = 0.024) and AST (OR 0.930, 95%CI 0.874–0.990; *p* = 0.023) were independent factors for IDE at 6-months follow-up.

**Table 4:** Association of variables with IDE at follow-up

Variables	No-adjusted Model				Age- and sex-adjusted model				Multivariate model*		
	OR	95%CI	p		OR	95%CI	p		OR	95%CI	p
**6 weeks post-LAAC**											
cauliflower	3.291	1.110–9.757	0.011		3.249	1.056–9.990	0.040		-	-	-
chicken-wing	0.322	0.105–0.989	0.048		0.334	0.106–1.053	0.061		-	-	-
HDL-C	9.687	1.297–72.330	0.023		8.394	1.037–67.966	0.046		15.109	1.637-139.478	0.017
ALT	0.968	0.137-1.000	0.049		0.967	0.936-1.000	0.047		-	-	-
AST	0.935	0.882–0.990	0.022		0.932	0.880–0.987	0.016		0.924	0.865–0.986	0.017
**6 months post-LAAC**											
cauliflower	3.182	1.085–9.331	0.035		3.236	1.043–10.039	0.042		-	-	-
HDL-C	7.887	1.156–53.789	0.035		6.971	0.939–51.733	0.058		11.015	1.365–88.896	0.024
AST	0.938	0.887–0.993	0.027		0.936	0.885–0.990	0.021		0.930	0.874–0.990	0.023

*Variables with *p* < 0.05 in univariate analysis were included in the multivariate model

Abbreviations: IDE, incomplete device endothelialization; OR, odds ratio; CI, confidence interval; HDL-C, High-density lipoprotein cholesterol; ALT, alanine aminotransferase; AST, aspartate transaminase

## Discussion

In this retrospective, single-center observational study, we evaluated the frequency of IDE at 6 weeks and 6 months after successful Watchman LAAC, and its impact on adverse clinical outcomes in Chinese AF patients. Our findings indicated that IDE was not uncommon in both 6 weeks and 6 months post-LAAC, particularly in patients with higher HDL-C and lower AST levels at baseline, although no significant difference in adverse cerebro-cardiac events was noted between incomplete and complete DE groups during 1-year follow-up.

### Incidence of IDE after LAAC

AF ablation has been incorporated into society guidelines as an important treatment modality based on results of several clinical studies comparing ablation with medical therapy [22]. Pulmonary vein isolation (PVI), as the cornerstone of AF, often arouse arrhythmia recurrence and subsequently other ablation strategies such as LAA isolation emerged [23]. Prior studies have displayed that the efficacy of combination LAA isolation with PVI in suppressing arrhythmias was superior to PVI alone, although, LAA isolation increases the risk of LAA thrombus [23, 24]. Most groups performed LAAC after a prior isolation to prevent thrombus formation inside the electrically altered LAA [23, 25]. And thus, LAAC has demonstrated the efficacy in preventing stroke and reducing bleeding events in comparison to long-term anticoagulation, but also used in AF ablation.

The positive outcomes from the PROTECT-AF and PREVAIL trials led to the approval of the Watchman device, the most commonly used LAAC device in the USA and Europe, to mitigate the risks of AF-associated thromboembolism [3, 26]. Anticoagulation is maintained after LAAC until device endothelialization, and current practice mandates the use of OAC strategies after Watchman placement, primarily based on animal evidence [4]. However, IDE has been frequently reported in humans beyond 6 months since 2012. For instance, Massarenti et al. [10] initially reported a case of IDE that persisted after 10 months of Watchman device implantation in a 74-year-old man with hereditary hemorrhagic teleangectasia. Subsequently, Sharma et al. [8] reported two cases of IDE after more than 1.5 years of Watchman LAAC. Remarkably, Mcivor et al. [27] discovered that a Watchman device was not endothelialized even at 3 years after the procedure. Such discrepancies in DE between animals and humans have prompted scholars to systematically investigate IDE in humans.

Presently, no standardized tests exist for assessing the degree of endothelialization in LAAC devices. In Granier et al.‘s study, IDE was defined as persistent permeability on LAA CTA without PDL on TEE during follow-up [28]. They found IDE in 14 out of 23 patients (61%) around 10 ± 6 months post-LAAC. Similarly, Lindner et al. [17] revealed that 20 out of 36 patients (56%) showed residual flow within the LAA without PDL, indicating IDE, at a median follow-up of 6 months post-LAAC. Applying the same IDE definition, our current study observed IDE in 48 (70.6%) and 46 (67.6%) of 68 Chinese AF patients with the Watchman device at the 6-week and 6-month follow-ups, respectively. Consistent with our findings, Xu et al. [29] also reported a 69% IDE rate using only LLA CTA imaging at the 6-month post-procedure mark; however, Xu et al. [29] categorized patients into CDE or IDE group without considering PDL. In contrast, Zhao et al. [30] reported a lower IDE rate of 8.3% at 6 months in 84 patients who underwent successful LAAC and AF ablation. Sivasambu et al. [31] observed IDE in 10 (21.8%) of 46 patients undergoing Watchman LAAC at 45 days during follow-up. Differences in IDE rates may be explained by prospective cohort studies indicating that over 40% of cases post-LAAC experience PDL [32]. Presence of PDL allows residual flow in the LAA, regardless of neo-endothelialization. Notably, Zhao et al. [30] and Sivasambu et al. [31] included patients with visible PDL in their studies, while our present study, as well as the investigations by Granier et al. [28] and Lindner et al. [17], excluded patients with PDL when exploring endothelialization levels. Given the limited current studies, further evidence is required to validate these findings, and additional methods and tests are essential for assessing neo-endothelialization after LAAC implantation.

### Risk factors for IDE

The potential predisposing factors for IDE post LAAC remain unclear, although some case reports have suggested that IDE may be attributed to an eccentric mitral regurgitation jet, diabetes, permanent AF, and larger implanted devices [8, 27]. In a prospective study by Xu et al. [29], persistent AF, increased LAA ostial diameter (> 23 mm vs. ≤ 23 mm), and left atrial (LA) size (> 44 mm vs. ≤ 44 mm) were identified as independent determinants for IDE in multivariate analysis. However, persistent AF, increased LAA ostial diameter, and LA size were studied only as binary categorical variables in their analysis. In our present study, LAA morphologies (cauliflower, chicken-wing) were initially observed to be significantly correlated with IDE in univariate analysis. Still, this significance disappeared after further adjustment for other confounding factors. Additionally, we did not find that age, AF type, LAA characteristics, or procedural factors were related to IDE. In line with our findings, the prospective observational study by Lindner et al. [17] and the retrospective study by Granier et al. [28] also failed to identify any predictive factors for IDE. Notably, clinical indicators were not considered in their studies. Whether clinical indicators serve as potential risk factors for IDE is a topic worth exploring.

In our current study, we also investigated the relationships of circulating clinical indicators before LAAC with endothelialization. A surprising observation by us was that higher HDL-C levels and lower AST levels were independently associated with IDE after LAAC. For a long time, HDL-C has been regarded as “good cholesterol” due to its robust inverse relationship with atherosclerotic cardiovascular disease [33, 34]. Recent research, however, has raised the causal nature of this relationship into question, with genetic studies failing to demonstrate an association between elevated HDL-C levels and reduced cardiovascular risk [35]. Moreover, a series of clinical trials also show no benefit of treatment using HDL-C-raising therapies [36, 37]. This discrepancy may be partly explained by the recent evidence that circulating HDL-C may become dysfunctional under pathological conditions [38, 39]. That is, impaired HDL-C is more prone to a proinflammatory property and remarkably reduces an anti-atherogenic vasoprotective function [40, 41], which is consistent with the evidence that HDL-C efflux capacity was strongly and inversely associated with coronary disease status [42]. In our current study, the mean HDL-C levels were 1.17 ± 0.33 mmol/L with up to 53% patients taking lipid-lowering drugs, which may make circulating high HDL-C levels be likely to dysfunctional to exhibit proinflammatory properties to delay the endothelization process. Additionally, AST is a traditional marker of myocardial injury. Recently, in a large cohort of community-dwelling patients with type 2 diabetes mellitus, Su et al. [43] demonstrated that patients with lower AST levels had an increased risk of cardiovascular mortality. Accordingly, they found that the risk of cardiovasular mortality decreased by 22% in the highest AST quintile compared with the lowest AST quintile after the multivariable analysis. A Mendelian randomization study in East Asians also illustrated that AST were inversely associated with cardiovascular disease [44]. Similar with them, we found that lower AST level was the independent factor for IDE after procedure. Nevertheless, data regarding the risk factors on IDE after implantation are sparse, large-scale prospective cohorts may need to further verify our findings.

### Clinical outcomes of 1-year follow-up

Despite the use of postprocedural anticoagulation, the potential for thrombus formation on the left atrial chamber side of the Watchman device remains associated with IDE [2]. The PROTECT-AF study [2] and the study by Kubo et al. [45] reported incidence rates of DRT at 4.2% (20/478) and 3.2% (4/119), respectively. However, in our present study with a limited sample size (*n* = 68), we did not identify any patients with DRT at 6 weeks and 6 months follow-up. This discrepancy may be attributed to our smaller sample size and variations in anticoagulant discontinuation practices. In our study, all patients strictly adhered to 45 days of anticoagulation followed by DAPT for 6 months without any bleeding events. In contrast, in Kubo et al.‘s study [45], among the four cases of DRT, one case had both warfarin and aspirin stopped before 45 days, and clopidogrel was discontinued before the 6-month point in another case. Consistent with the results by Xu et al. [29] at 6-month clinical follow-up, we also found no significant difference in adverse cerebro-cardiac events between IDE and CDE groups at the 1-year follow-up. No significant differences of MACCE between CDE and IDE groups in our study may be also related to the facts that 1-year follow-up was a little short and all patients in our study strictly implemented medical therapies after LAAC. Further large-scale investigations with longer follow-up periods are needed to better understand the consequences of IDE based on the limited data available.

### Strengths and limitations

The strengths of our present study as follows. To the best of our knowledge, it is the first study to systematically investigate the prevalence and potential predictors of IDE using the clinical data of before and post operation in Chinese AF patients undergoing LAAC. Our results strongly indicated that IDE was common at the 6 weeks and 6 months after post-LAAC, and patients with higher HDL-C and lower AST levels at baseline had prominently higher risk for IDE, which suggested that the combination of the clinical indicators before operation and TEE, LAA CTA after operation may be a better method for the detection of IDE, and helpfully improved clinical outcomes. In addition, we will further develop an artificial intelligence (AI)-based approach for the prognosis assessment and postoperative treatment plans of LAAC by the baseline clinical data of patients before the operation, based on the development and effectiveness of artificial intelligence (AI) on the management of coronary artery disease and AF in recent decades [46]. For example, Jiang et al. [47] developed an AI-enabled ECG algorithm to predict the risk of recurrence in patients with paroxysmal AF after catheter ablation. However, some limitations were needed to be mentioned in our present study. Firstly, our sample size looked like relatively small, but participants identified as non-PDL after performing both TEE and LAA CTA at follow-up were entered into our final analysis. Furthermore, all the patients in our present study were using Watchman devices to avoid the differences among different device types. Secondly, the study participants were from a single center and represented the Chinese Han population, limiting the generalizability of our results to other ethnic groups. Thirdly, recent studies have shown that a staggering 37% of all AF patients have underlying dementia and AF is independently associated with other neurological disorders, including cognitive impairment and dementia [48, 49, 50]. As the related data was not collected in our follow-up, and thus, it is worthy of studying the associations of IDE with cognitive impairment and dementia at follow-up in more multi-center studies with longer follow-up.

## Conclusions

In conclusion, our study found that neo-endothelialization existed incomplete in the majority Chinese AF patients even at the 6-month follow-up after successful LAAC therapy, particularly accompanying with higher HDL-C and lower AST levels. Therefore, in clinical practice, the combined application of the clinical indicators before operation and TEE, LAA CTA after operation should be advocated to identify IDE after LAAC in order to properly extend the anticoagulation time to prevent the cardio-cerebrovascular complication. Moreover, patients with higher HDL-C and lower AST levels should be enhanced the monitoring.

## Data Availability

All data generated or analysed during this study are included in this published article.
